# Humanized *Dsp* ACM Mouse Model Displays Stress-Induced Cardiac Electrical and Structural Phenotypes

**DOI:** 10.3390/cells11193049

**Published:** 2022-09-29

**Authors:** Tyler L. Stevens, Heather R. Manring, Michael J. Wallace, Aaron Argall, Trevor Dew, Peter Papaioannou, Steve Antwi-Boasiako, Xianyao Xu, Stuart G. Campbell, Fadi G. Akar, Maegen A. Borzok, Thomas J. Hund, Peter J. Mohler, Sara N. Koenig, Mona El Refaey

**Affiliations:** 1 Frick Center for Heart Failure and Arrhythmia Research, The Dorothy M. Davis Heart and Lung Research Institute, The Ohio State University Wexner Medical Center, Columbus, OH 43210, USA; 2 Department of Physiology and Cellular Biology, The Ohio State University College of Medicine and Wexner Medical Center, Columbus, OH 43210, USA; 3 Comprehensive Cancer Center, The Ohio State University College of Medicine and Wexner Medical Center, Columbus, OH 43210, USA; 4 Department of Surgery, Division of Cardiac Surgery, The Ohio State University College of Medicine and Wexner Medical Center, Columbus, OH 43210, USA; 5 Department of Biomedical Engineering, Yale University, New Haven, CT 06520, USA; 6 Department of Cellular and Molecular Physiology, Yale School of Medicine, New Haven, CT 06520, USA; 7 Department of Internal Medicine, Section of Cardiovascular Medicine, Yale School of Medicine, New Haven, CT 06520, USA; 8 Biochemistry, Chemistry, Engineering, and Physics Department, Commonwealth University of Pennsylvania, Mansfield, PA 16933, USA; 9 Department of Biomedical Engineering, The Ohio State University, Columbus, OH 43210, USA; 10 Department of Internal Medicine, Division of Cardiovascular Medicine, The Ohio State University College of Medicine and Wexner Medical Center, Columbus, OH 43210, USA

**Keywords:** desmoplakin, arrhythmogenic cardiomyopathy, arrhythmia, mouse model, intercalated disc, cardiac stress

## Abstract

Arrhythmogenic cardiomyopathy (ACM) is an inherited disorder characterized by fibro-fatty infiltration with an increased propensity for ventricular arrhythmias and sudden death. Genetic variants in desmosomal genes are associated with ACM. Incomplete penetrance is a common feature in ACM families, complicating the understanding of how external stressors contribute towards disease development. To analyze the dual role of genetics and external stressors on ACM progression, we developed one of the first mouse models of ACM that recapitulates a human variant by introducing the murine equivalent of the human R451G variant into endogenous desmoplakin (*Dsp^R451G/+^)*. Mice homozygous for this variant displayed embryonic lethality. While *Dsp^R451G/+^* mice were viable with reduced expression of DSP, no presentable arrhythmogenic or structural phenotypes were identified at baseline. However, increased afterload resulted in reduced cardiac performance, increased chamber dilation, and accelerated progression to heart failure. In addition, following catecholaminergic challenge, *Dsp^R451G/+^* mice displayed frequent and prolonged arrhythmic events. Finally, aberrant localization of connexin-43 was noted in the *Dsp^R451G/+^* mice at baseline, becoming more apparent following cardiac stress via pressure overload. In summary, cardiovascular stress is a key trigger for unmasking both electrical and structural phenotypes in one of the first humanized ACM mouse models.

## 1. Introduction

Arrhythmogenic cardiomyopathy (ACM) is characterized by fibro-fatty replacement of ventricular myocardium and increased propensity to fatal arrhythmias [1,2]. In an early pre-symptomatic ’concealed phase’, structural hallmarks of ACM are unidentifiable, yet the risk for life-threatening ventricular arrhythmias and sudden cardiac death (SCD) is apparent, with up to 50% of index cases experiencing SCD as the first clinical manifestation [3,4,5]. Incomplete penetrance further complicates the disease progression, as individuals with the same disease-causing variant may experience significant differences in disease symptoms and severity [6].

Despite the large variance in genetic causes and environmental factors, the majority of ACM cases with a disease-causing variant (~85–90%) are linked to variants in desmosomal genes [7,8,9]. Desmoplakin (*DSP*) has an N-terminal mutational ‘hotspot’ for ACM variants [10]. Indeed, previous work identified select variants in this region have sensitivity to calpain-mediated degradation [11]. We further focused our work on one calpain-sensitive DSP variant (p.R451G). Using induced pluripotent stem cells (iPSCs) from ACM patients within this single kindred, we identified reduced post-translational expression of the full length protein [11]. While this was important in identifying how multiple DSP variants share a specific mechanism of pathogenicity, a major limitation of these models is understanding phenotypic progression. Furthermore, incomplete penetrance is frequently observed in ACM families, and multiple carriers of the R451G variant fail to display any ACM phenotype.

Nearly every ACM animal model relies on a knock-out or transgenic knock-in system [12,13,14,15,16,17,18], with most models failing to explain phenotypic discrepancies between individuals carrying the same variant. Here, we report the generation of one of the first ACM disease models endogenously expressing the human equivalent of a point variant in *DSP* using CRISPR/Cas9 gene editing. *Dsp^R451G/+^* mice displayed decreased full length DSP protein level with no differences at the transcript level. Attempted generation of a homozygous variant model (*Dsp^R451G/R451G^*) led to the discovery of embryonic lethality prior to E10, supporting severe detrimental effects associated with the variant. *Dsp^R451G/+^* mice displayed no changes in cardiac structure or function throughout adulthood in the absence of stress. Additionally, *Dsp^R451G/+^* mice did not show baseline arrhythmias or other electrical abnormalities at baseline. However, *Dsp^R451G/+^* mice progressed to heart failure at an earlier timepoint following pressure overload with increased chamber dilation and reduced fractional shortening. Furthermore, *Dsp^R451G/+^* mice displayed increased prevalence and severity of prolonged arrhythmias following catecholaminergic challenge. At the molecular level, altered localization of connexin-43 (Cx43) was noted in *Dsp^R451G/+^* hearts, and localization patterns were further disrupted following cardiac stress. These findings highlight the role of cardiac stress in ACM disease phenotypes and severity. 

## 2. Materials and Methods

### 2.1. Animal Studies

The R464G (murine equivalent of human R451G) variant was introduced into endogenous *DSP* (whole body) in a C57BL/6J background using a CRISPR/Cas9 system guided by single-stranded oligodeoxynucleotides. The incorporation of this variant introduced a restriction enzyme site for BstNI. Sanger sequencing was used to identify founders capable of starting the R451G line. All experimental mice were backcrossed into a pure C57BL/6 background and wildtype littermates were used as controls throughout the manuscript. Genotypes were confirmed using PCR with restriction enzyme digest and Sanger sequencing. A full list of primers used can be found in Supplementary Table S1. Age-matched male and female mice were used throughout the studies to analyze potential sex-based differences (not observed in this study, so male and female findings were combined). Adult mice were studied at ~3 months of age, with older mice characterized at ~6 months of age.

### 2.2. Embryo Isolations

*Dsp^R451G/+^* X *Dsp^R451G/+^* crosses we set up for <16 h to identify the timepoint of *Dsp^R451G/R451G^* embryonic lethality. Females were examined for a vaginal plug, and males were immediately removed from the breeding cage. Female mice were aged for appropriate embryonic stage development prior to isolation (E4, E6, E8, E9.5, E11.5, E13, and E16). Embryos were isolated, separated from the placenta, and imaged to identify any abnormalities. Tissue samples/yolk sacs were digested and sent for sequencing to verify genotypes. 

### 2.3. Transverse Aortic Constriction (TAC) Surgeries

All mice used for surgery were aged to ~3 months of age, weighing between 22–25 g. Surgeries were conducted as previously described [19]. The surgeon was blinded to genotype. TAC mice had baseline measurements recorded within 48 h of surgery. Measurements were recorded at two, four, six, eight, ten, and twelve weeks post-surgery. Mice that died due to procedural complications of the TAC surgery either during, or within 72 h of the surgery were only included in baseline statistics (*n* = 10 per genotype), and were not included in the Kaplan–Meier curve [20,21].

### 2.4. Telemetry Surgeries

Telemetry implantation procedures were performed as described [19]. 

### 2.5. Echocardiography

All mice were anesthetized using 2.0% isoflurane in 95% O_2_ and 5% CO_2_ at ~0.8 L/min. Oxygen administration was continued with ~1% isoflurane throughout the recording process. Following hair removal, recordings were collected of the left ventricle (LV) in a long axis view, with contractile parameters and chamber dimensions being recorded using the M-mode function in the short axis view. Parameters analyzed in short axis M-mode include: left ventricular internal diameter at systole and diastole (LVIDs/d), interventricular septal end systole and diastole (IVSs/d), end systolic volume (ESV), end diastolic volume (EDV), left ventricular posterior wall end systole and diastole (LVPWs/d) ejection fraction (EF%), fractional shortening (FS%), and heart rate (HR-BPM). Heart rate was monitored throughout imaging and recordings associated with a heart rate <400 bpm were excluded.

### 2.6. Electrocardiograms

Telemeter ECG recordings were conducted using Ponemah Physiology Platform version 5.2 acquisition software (Version 5.20 for Windows, Data Sciences International, St. Paul, MN, USA), ten minutes prior, and 30 min after 2 mg/kg intraperitoneal epinephrine injections. All ECG files were analyzed using LabChart 8 software (ADInstruments, Sydney, Australia). Intervals analyzed include RR interval, PR interval, P duration, QRS interval, QT/QTc interval (Mitchell et al. normalization [22]), Tpeak-Tend interval, and heart rate. Surface ECG analysis was only conducted on mice post-TAC surgery, with ten minute recordings being conducted at baseline, four, eight, and twelve weeks post-surgery. Arrhythmic events analyzed include premature ventricular contractions (PVC), atrioventricular (AV) block, non-sustained ventricular tachycardia (NSVT- minimum of 3 consecutive PVC events lasting < 1 s [23]), ventricular tachycardia (VT, >1 s event), and bigeminy (regular sinus beat followed by PVC, at least 3 consecutive occurrences [24]).

### 2.7. Immunoblotting

All murine hearts were washed in cold PBS to remove blood, and immediately placed in either chilled 1% SDS-1% BME lysis buffer with protease inhibitor (PI) for standard protein isolation, or PhosphoSafe extraction reagent (Millipore-71296, Millipore Sigma, St. Louis, MO, USA) with PI for evaluating phosphorylated targets (pCx43). Samples were homogenized using bead homogenization (Precellys 24, Bertin Instruments, Rockville, MD, USA). Following protein quantification, 40 µg of protein was used to prepare each sample. Samples were electrophoresed on either a NuPAGE 3–8% Tris-Acetate gel (Thermo-Fisher Scientific, Waltham, MA, USA) for >250 kD proteins (DSP), or Blot 4–12% Bis-Tris Plus gel (Bio-Rad, Hercules, CA, USA) for <250 kD proteins. Membranes were blocked in 5% NFDM or Casein (Thermo-Fisher Scientific, Waltham, MA, USA) for one hour, depending on the protein of interest. Following blocking, membranes were incubated with a primary antibody overnight at 4 °C and then incubated with secondary antibody for two hours at room temperature. Densitometry was performed using Image J software (NIH, Bethesda, MD, USA).

### 2.8. Immunofluorescence 

Whole hearts were isolated from ~3-month-old, (plakoglobin and connexin-43 staining), ~6-month-old (DSP staining), and TAC surgery *Dsp*^R451G/+^ mice and control littermates for endpoint studies. All hearts were submerged in OCT, oriented for four chamber view sectioning, and frozen using liquid nitrogen vapors. Hearts were sectioned at The Ohio State University’s comparative pathology and mouse phenotyping core laboratory. Adult ventricular myocytes were prepared as previously described [25]. Isolated cells were fixed and stored in 70% EtOH at −20 °C. Both isolated ventricular myocytes and cryosections were blocked for two hours in fish blocking solution (3% fish gel, 0.75% Triton X100 [10%], and 1% DMSO), followed by overnight incubation in primary antibody at 4 °C. Samples were washed the following day and incubated in secondary antibody at room temperature for two hours. For negative controls, replicate sections/cells were only incubated with secondary antibodies for two hours. Sections and isolated cells were covered with Vectashield imaging medium with DAPI (Vector Laboratories, Newark, CA, USA), and coverslips were applied and sealed. Images were obtained using a confocal microscope (LSM 510 Meta, Zeiss, Oberkochen, Germany) Images were collected using identical confocal protocol settings at room temperature, and the observer was blinded to the genotype. Quantification of signal intensity was performed using image J software. Area of overlap between two signals was calculated using ROI manager under Image J software. All calculations were normalized to control. A full list of antibodies used can be found in Supplementary Table S2. 

### 2.9. Transcript Analysis

Total RNA was isolated from ~3 month old hearts. Hearts were immediately placed in Trizol and bead homogenized at 4 °C. RNA concentrations and purity were analyzed using a Nanodrop 1000 (Nanodrop Technologies, Thermo-Fisher Scientific, Wilmington, DE, USA). Purified mRNA (2 µg) underwent reverse transcription using the SuperScript IV VILO Master Mix (Thermo-Fisher Scientific, Waltham, MA, USA) with ezDNAase enzyme protocol (Invitrogen, Thermo-Fisher Scientific, Carlsbad, CA, USA). qPCR was performed on cDNA examining *Dsp* levels, using *Hprt* as a comparable control. 

### 2.10. Tissue Histology and Staining

Whole hearts were isolated from ~6 month old *Dsp^R451G/+^* and control littermates, as well at end timepoints of ~3 month old mice following telemetry surgery and recordings. Hearts were rinsed in cold PBS, rocked in Krebs–Henseleit solution for 30 min, fixed in 10% formalin overnight, and moved into 70% EtOH until paraffin embedding. Heart sections at 5 µm thickness were obtained for four chamber views. Heart processing, sectioning, and H&E staining were performed at the Ohio State University’s Wexner Medical Center human pathology/histology core. Trichrome staining was performed on sections using a Masson’s Trichrome 2000 Stain Kit (KTMTR2, American Mastertech Scientific, StatLab, Lodi, CA, USA). Fibrosis analysis of trichrome images was modified from Gratz et al. as previously described [26]. 

### 2.11. Statistics

Data are presented as mean ± S.E.M. For the comparison of two groups, we performed unpaired two-tailed Student’s *t*-test when data passed normality test (Shapiro–Wilk normality test). When data failed this normality test, a Mann–Whitney test was performed to compare the groups and box and whisker were used to display data. Differences were considered significant at a *p* < 0.05. Statistical analysis was performed using GraphPad Prism (Version 7.01 for Windows, GraphPad Software, San Diego, CA, USA). 

## 3. Results

### 3.1. Homozygous DSP p.R451G Knock-In Mice Display Embryonic Lethality

**Figure 1:**
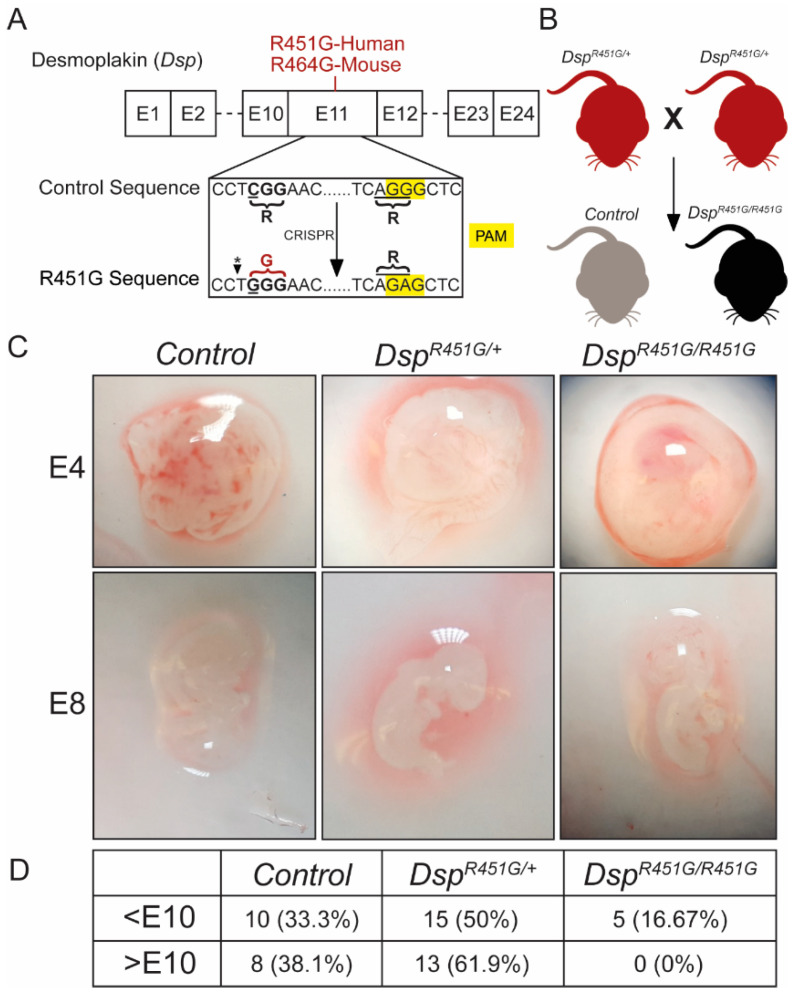
Schematic overview of the R451G mouse model. (**A**) Amino acid substitution occurs at residue R464, the murine equivalent of the human R451G variant. Alteration at the PAM site results in no changes within the coding sequences. * Introduction of the R451G variant results in the generation of a restriction enzyme cut site for BstNI (CC^∨^WGG) that was utilized for genotyping. (**B**) Schematic for the generation of *Dsp^R451G/R451G^* mice and control littermates after backcrossing is completed. (**C**) Embryonic isolations at time points <E10 identified viable *Dsp^R451G/R451G^* embryos. (**D**) Breakdown of timepoints and genotypes from isolated embryos from *Dsp^R451G/+^ X Dsp^R451G/+^*, with no viable *Dsp^R451G/R451G^* embryos isolated after E10.

The N-terminus of DSP is the location of multiple human ACM variants [10]. The R451G variant in this region is of interest due to its presence and variable phenotype in a large kindred, as well as the linkage of this variant to calpain-dependent proteolysis in vitro [11]. To assess this phenotypic variability, we generated a mouse model expressing endogenous DSP^R451G^ (Figure 1A). Mice heterozygous for the DSP p.R451G variant (*Dsp^R451G/+^*) were bred to study *Dsp^R451G/R451G^* mice in comparison to control littermates (Figure 1B). *Dsp^R451G/R451G^* mice were embryonic lethal, and the ratio of *Dsp^R451G^*^/+^ to control littermates was approximately 2:1, the expected Mendelian ratio of offspring assuming *Dsp^R451G/R451G^* mice were not viable. Embryonic isolations were performed from *Dsp^R451G/+^* X *Dsp^R451G/+^* breeding pairs, and viable *Dsp^R451G/R451G^* embryos were identified prior to E10, including E4 and E8 (Figure 1C). Verification of genotyping was performed utilizing Sanger sequencing to confirm the nucleotide change at the mutation site as well as the PAM sequence (Supplementary Figure S1). After E10, no viable *Dsp^R451G/R451G^* embryos were detected (Figure 1D). Thus, *Dsp^R451G/R451G^* mice are embryonic lethal prior to E10. As a result of this lethality, only *Dsp^R451G/+^* mice were evaluated for cardiac phenotypes. These heterozygotes mirror the autosomal dominant inheritance pattern observed in the original R451G patient family [11]. 

**Figure 2:**
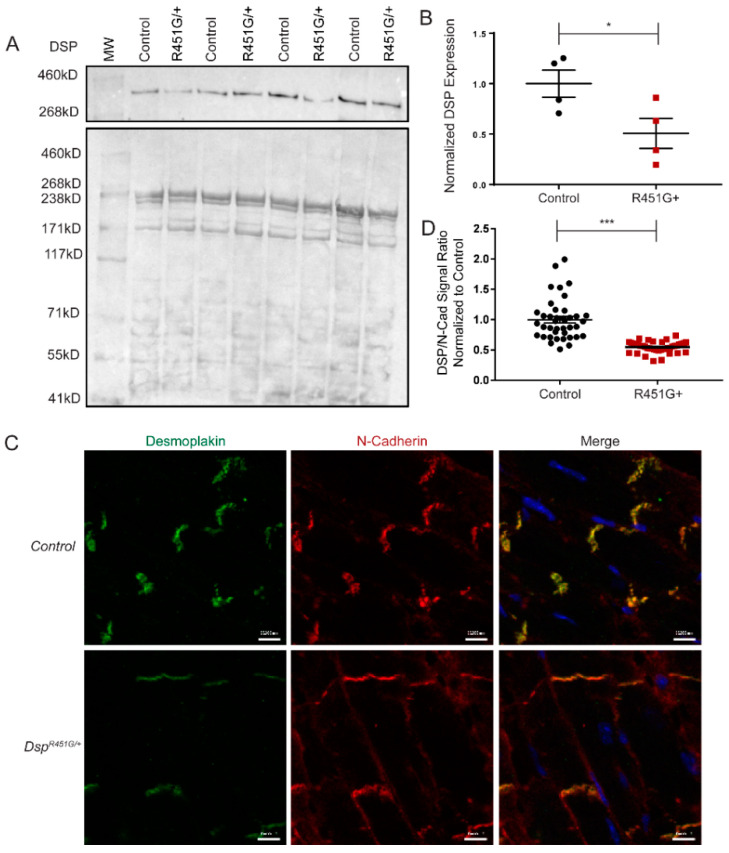
*Dsp^R451G/+^* mice display a reduction in DSP protein expression. (**A**) Representative immunoblot of heart lysates from *Dsp^R451G/+^* and control littermates isolated at 6 months of age, comparing full length DSP levels (*n* = 4 per group). Ponceau staining included. (**B**) Quantification of immunoblotting of DSP normalized to Ponceau staining. Stats performed using Student’s *t*-test * *p* < 0.05. (**C**) Immunostaining of cardiac cryosections examining DSP localization and expression patterns at the intercalated disc, using N-cadherin as a control marker (*n* = 4 per genotype). Scale bars represent 10 µm. (**D**) Quantification of DSP/N-Cad signal intensity ratios. *n* = 4 animals per genotype, *n* = 10 IDs analyzed per animal; stats performed using Student’s *t*-test *** *p* < 0.001.

Previous data from R451G human studies revealed a loss of full-length DSP protein that occurred post-translationally [11]. Heart lysates from control and *Dsp^R451G^*^/+^ littermates were analyzed at ~6 months of age. *Dsp^R451G/+^* mice displayed an approximate 50% reduction in full-length DSP protein in comparison to control littermates (Figure 2A,B). This was further confirmed in lysates from ~1 month and 3 month hearts. All lysates displayed a significant, near 50% reduction in DSP expression (Supplementary Figure S2A,B). We did not observe any small fragments of DSP unique in the *Dsp^R451G^*^/+^ heart lysates. mRNA levels of *Dsp* were unchanged between *Dsp^R451G^*^/+^ and control hearts, indicating the reduction in DSP expression occurred post-translationally (Supplementary Figure S2C). To ensure there were no potential dominant-negative effects from the R451G DSP variant, immunostaining of heart sections isolated at ~6 months of age was performed to analyze the localization of DSP. While *Dsp^R451G^*^/+^ heart tissue showed a significant reduction in DSP signal at the intercalated disc (ID) in relation to the control marker N-cadherin, we did not observe DSP mislocalization from the ID (Figure 2C,D). In summary, our findings support that DSP p.R451G is post-translationally unstable but does not impact the localization of DSP from the control allele.

### 3.2. Dsp^R451G/+^ Mice Do Not Display Changes in Cardiac Structure or Function at Baseline

**Figure 3:**
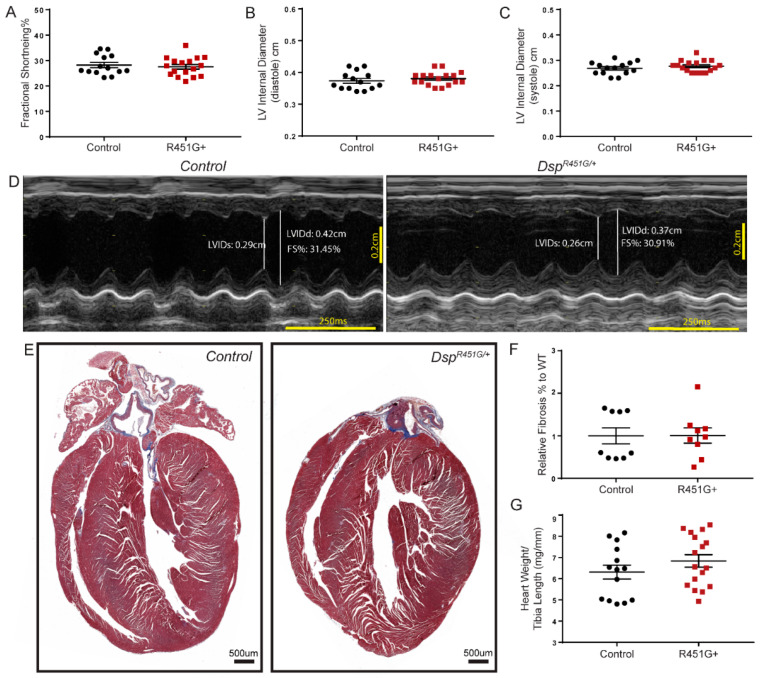
*Dsp^R451G/+^* mice display no structural changes at baseline. (**A**) Baseline echocardiography measurements conducted on 6-month control and *Dsp^R451G/+^* mice and littermates comparing fractional shortening, (**B**) LVIDd, (**C**) and LVIDs (control *n* = 14, *Dsp^R451G/+^ n* = 17). (**D**) Representative M-mode echocardiography recordings from control and *Dsp^R451G/+^* mice performed at 6 months. (**E**) Masson’s trichrome staining performed on 6 month old control and DSP^R451G/+^ mice littermates. (**F**) Relative fibrosis levels calculated from Masson’s trichrome staining (*n* = 9 per genotype). (**G**) Heart weight to tibia length ratio isolated following echocardiography experiments (control *n* = 14, *Dsp^R451G/+^ n* = 17) Stats performed using Student’s *t*-test.

Structural abnormalities have been previously observed in select individuals harboring the DSP p.R451G variant, where the majority of patients experience left or biventricular dysfunction [11]. We evaluated six-month *Dsp^R451G/+^* mice for potential structural or functional cardiac phenotypes. We observed no changes in cardiac function as assessed by measuring fractional shortening (FS%), or left ventricular internal diameter (LVID; both systolic and diastolic; Figure 3A–D). A summary of all baseline echocardiographic data is noted in Supplementary Table S3. We observed no signs of increased fibro-fatty infiltration in *Dsp^R451G^*^/+^ hearts (Figure 3E,F). Finally, *Dsp^R451G^*^/+^ mice displayed no changes in heart weight to tibia length ratio (Figure 3G). 

### 3.3. Dsp^R451G/+^ Mice Do Not Display Spontaneous Arrhythmias at Baseline

Electrical defects commonly precede structural abnormities in ACM [4,27]. Therefore, we evaluated *Dsp^R451G^*^/+^ mice for arrhythmia susceptibility in the absence of structural defects. Similar to control littermates, no baseline electrical abnormalities were noted in *Dsp^R451G^*^/+^ mice (Supplementary Figure S3A). However, we observed trends toward a prolonged QTc (*p* = 0.0914) and T-peak to T-end (*p* = 0.0609), with no changes in heart rate, suggesting potential defects in repolarization (Supplementary Figure S3B,C, Supplementary Table S4).

### 3.4. Dsp^R451G/+^ Mice Display Accelerated Heart Failure Following Pressure Overload

**Figure 4:**
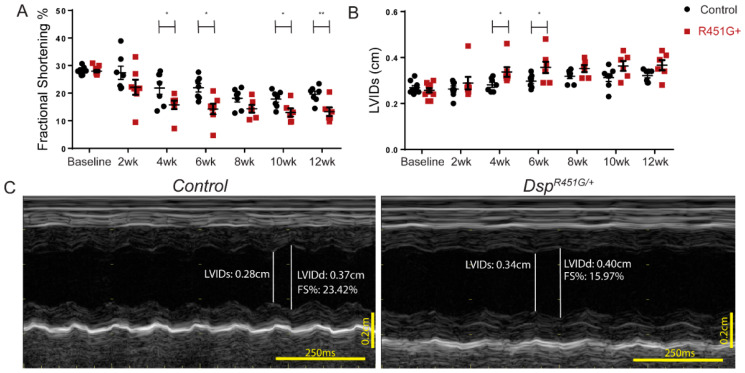
*Dsp^R451G/+^* mice display structural changes post-TAC surgery. (**A**) Comparison of fractional shortening (**B**) and LVIDs between *Dsp^R451G/+^* mice and control littermates post-TAC surgery. Measurements taken every 2 weeks after surgery (control *n* = 7, *Dsp^R451G/+^ n* = 6–7 post-surgery). Stats performed using Student’s *t*-test within each timepoint * *p* < 0.05, ** *p* < 0.01. (**C**) Representative M-mode echocardiography recordings from *Dsp^R451G/+^* and control littermates 4 weeks post-TAC surgery.

To evaluate the response of cardiac output following chronic cardiac stress, transverse aortic constriction (TAC) surgeries were performed on *Dsp^R451G^*^/+^ mice to induce pressure overload. Surgeries were performed at ~3 months of age, and echocardiography measurements were acquired at baseline and every two weeks post-surgery for twelve weeks. Four weeks post-TAC, *Dsp^R451G^*^/+^ mice displayed a significant reduction in FS% compared to control littermates (Figure 4A: Control FS% = 21.84%, *Dsp^R451G^*^/+^ FS% = 15.77%; *p* = 0.0448). Interestingly, *Dsp^R451G^*^/+^ mice also demonstrated an increase in LVIDs as early as four weeks post-TAC when compared to the control littermates (Figure 4B,C: Control LVIDs = 0.281 cm, *Dsp^R451G^*^/+^ LVIDs = 0.337 cm; *p* = 0.0368). These parameters remained significant or trended towards significance throughout the study until the ending timepoint. Diastolic measurements at four weeks post-TAC, including LVIDd (*p* = 0.0520) and end diastolic volume (*p* = 0.0783), showed a trending increase in *Dsp^R451G^*^/+^ mice (Supplementary Figure S4A,B). There was a trending increase to end systolic volume in *Dsp^R451G^*^/+^ mice 4 weeks post-surgery compared to control littermates (*p* = 0.0810, Supplementary Figure S4C), with ejection fraction (EF%) trending or significantly decreased in *Dsp^R451G^*^/+^ mice at each timepoint starting at 4 weeks post-surgery (Supplementary Figure S4D). No significant changes in overall survival, or heart weight/tibia length ratio were observed throughout the study (Supplementary Figure S4E,F). 

At baseline, *Dsp^R451G^*^/+^ mice displayed a trending, but not statistically significant increase in QTc interval as well as T-peak to T-end interval (Supplementary Figure S3B, Supplementary Table S4). To evaluate how this electrical phenotype was altered in response to pressure overload, ECG recordings were acquired from anesthetized mice every four weeks post-TAC. The QTc interval was significantly prolonged at four weeks post-TAC in *Dsp^R451G^*^/+^ mice, and the T-peak to T-end showed a trending increase that did not reach significance (*p* = 0.0584; Supplementary Figure S5A–C). No other changes in electrical parameters were noted following surgery (Supplementary Figure S5D–F). 

ECG morphological changes were also analyzed, and at four weeks post-TAC, control mice displayed minimal changes in the T-wave morphology, whereas *Dsp^R451G^*^/+^ mice showed more severe T-wave inversion (Supplementary Figure S6). Notably, T-wave inversion is a clinical diagnostic criterion of ACM and suggests a significant risk of SCD [28,29]. Lastly, *Dsp^R451G^*^/+^ mice displayed a fragmented pattern in the QRS (fQRS) structure, another diagnostic parameter for ACM, that was not observed in the control littermates (Supplementary Figure S6). Taken together, *Dsp^R451G^*^/+^ mice displayed no changes in cardiac structure or function at baseline. However, *Dsp^R451G^*^/+^ mice exhibited reduced cardiac function, LV dilation, and signs of accelerated heart failure phenotypes following pressure overload. 

### 3.5. Dsp^R451G/+^ Mice Display Stress-Induced Arrhythmias

**Figure 5:**
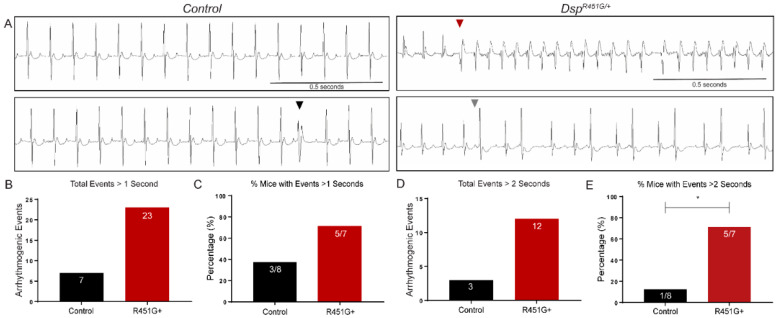
*Dsp^R451G/+^* mice display arrhythmias following catecholaminergic challenge. (**A**) Representative ECG recordings of control and *Dsp^R451G/+^* mice littermates (~3 months of age) following 2 mg/kg epinephrine. Arrowheads point to identified arrhythmic events, including isolated PVCs (Black), ventricular tachycardia (red), and bigeminy (gray). Scale bars represent 500 ms. (**B**) Arrhythmogenic events that exceeded 1 s per group. (**C**) Percentage of mice in each genotype with at least 1 event that exceeded 1 s. (**D**) Total number of arrhythmogenic events longer than 2 s. (**E**) Percentage of mice in each genotype with at least 1 event that exceeded 2 s. Stats performed using Fisher’s exact test * *p* < 0.05 (control *n* = 8, *Dsp^R451G/+^ n* = 7).

To determine if *Dsp^R451G^*^/+^ mice were prone to inducible arrhythmias prior to structural remodeling, we investigated the impact of catecholamines on cardiac electrical activity. As expected, following 2.0 mg/kg epinephrine, control mice displayed limited arrhythmogenic events. In contrast, *Dsp^R451G/+^* mice displayed prolonged and severe arrhythmogenic events, including example traces of ventricular tachycardia (VT) and bigeminy (Figure 5A–C). Additionally, a greater proportion of *Dsp^R451G/+^* mice (71.4%) experienced arrhythmogenic events that exceeded two seconds compared to control littermates (12.5%, *p* = 0.0406; Figure 5D–E). *Dsp^R451G/+^* mice also displayed a significant increase in atrioventricular block (AV) block events, as well as a trending increase in premature ventricular contractions (PVCs) (*p* = 0.144; Supplementary Figure S7A,B). No significant differences in rate of occurrence were observed in other types or arrhythmic events (Supplementary Figure S7C,D). Taken together, *Dsp^R451G/+^* mice were prone to more frequent and prolonged arrhythmias following catecholaminergic stimulation in the absence of a structural phenotype.

### 3.6. Dsp^R451G/+^ Mice Display Normal Localization of Key ID Proteins but Altered Distribution of Cx43

**Figure 6:**
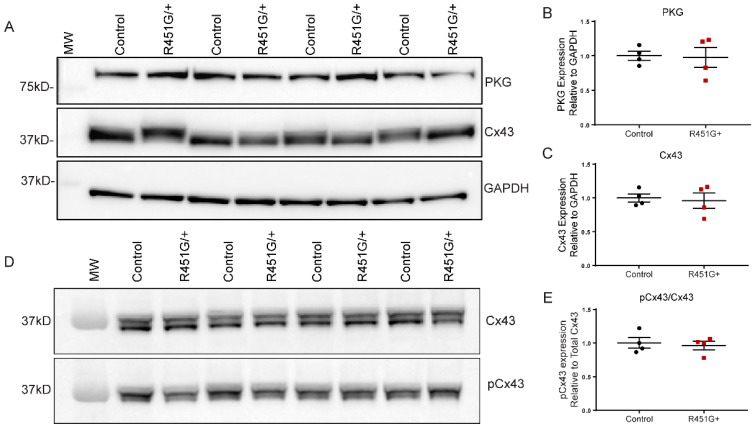
*Dsp^R451G/+^* mice display no changes in expression of key ID proteins. (**A**) Immunoblots of heart lysates from *Dsp^R451G/+^* mice and control littermates isolated at 3 months of age, evaluating key ID markers PKG and Cx43. (**B**,**C**) Quantification of PKG and Cx43 protein expression relative to GAPDH. (**D**) Immunoblots probing for pCx43 (Ser368) and Cx43. (**E**) Quantification of pCx43 protein expression compared to total Cx43. Stats performed using Student’s *t*-test (*n* = 4 per genotype).

Based on the critical role of DSP in cardiac function, we hypothesized that the R451G missense variant would cause reduced expression and/or altered localization of key DSP binding partners in mice. Initial targets of interest included Cx43 and plakoglobin (PKG), as these proteins were shown to be frequently altered in ACM models and patients [13,14,30,31,32]. R451G+ biopsy samples and human iPSCs previously identified Cx43 mislocalization, decreased Cx43 protein expression, and increased phosphorylation at Cx43-S368 [11]. Expression levels of PKG and Cx43 were not different between *Dsp^R451G/+^* mice and their control littermates (Figure 6A–C). Further, pCx43/Cx43 ratios were not altered as well (Figure 6D,E). We also examined additional targets including integrin-β1D, a protein reduced in many ACM cases with reduced DSP expression [33], as well as plakophillin-2 (PKP2) and β-catenin, ACM-linked proteins that localize at the ID [34,35]. Notably, immunoblotting revealed no changes in expression patterns for integrin-β1D, PKP2, or β-Catenin (Supplementary Figure S8A–C).

**Figure 7:**
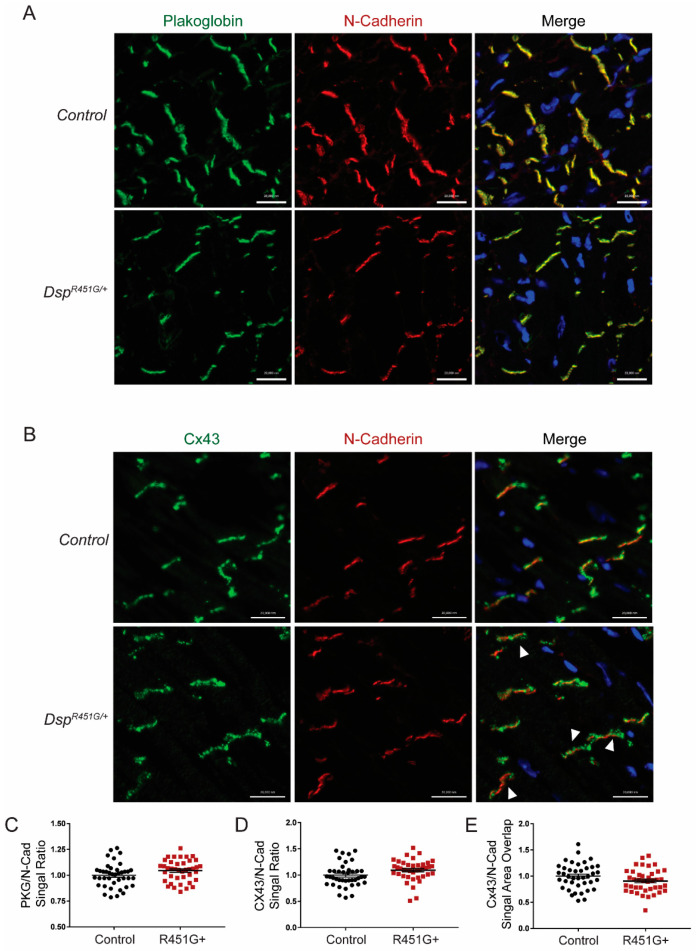
*Dsp^R451G/+^* mice display no changes in key ID protein localization. (**A**) Immunofluorescent (IF) images of cardiac cryosections examining plakoglobin, N-cadherin co-stain used as a control marker. (**B**) Staining for Cx43 localization patterns at the ID, with arrowheads pointing to ID with punctate Cx43 expression at the disc in comparison to control marker N-cadherin. Scale bars represent 20 µm. *n* = 4 per genotype. (**C**) Quantification of PKG/N-Cad (**D**) and Cx43/N-Cad signal intensity ratios. (**E**) Quantification of Cx43/N-Cad area overlap. Stats performed using Student’s *t*-test (*n* = 4 animals per genotype, *n* = 10 IDs analyzed per animal).

Consistent with expression data, we observed no changes in PKG localization, as expression was uniform at the disc when compared to control marker N-cadherin (Figure 7A). However, we did observe minor, but consistent alterations of Cx43 at the ID when compared to the control heart tissue sections. Specifically, Cx43 displayed a pronounced punctate expression pattern in *Dsp^R451G/+^* tissue when compared to control sections (Figure 7B). In *Dsp^R451G/+^* mice, neither PKG nor Cx43 expression at the ID was reduced in cryosections when compared to control marker N-cadherin (Figure 7C,D). However, when compared to control mice, *Dsp^R451G/+^* tissue displayed a trending decrease in Cx43 overlap with N-cadherin by ~10% (Figure 7E, *p* = 0.076). In summary, while displaying minor alterations in Cx43 localization, *Dsp^R451G/+^* mice had normal expression and distribution of key DSP-associated binding partners at baseline.

**Figure 8:**
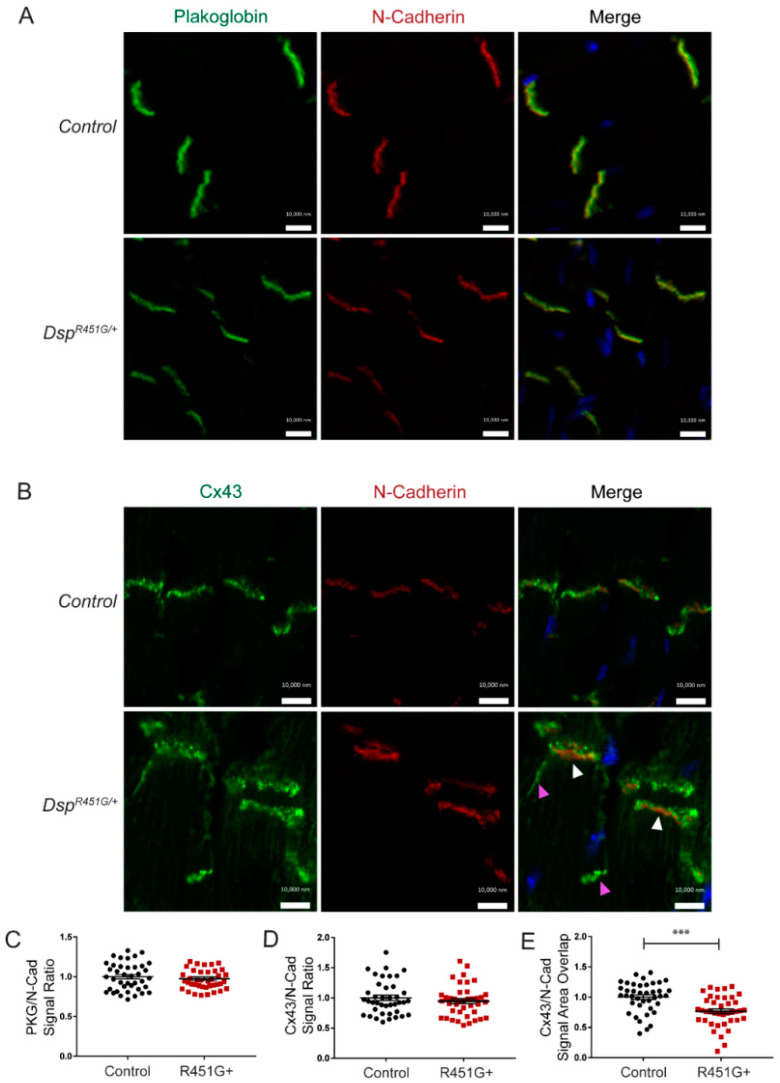
*Dsp^R451G/+^* mice display enhanced mislocalization of Cx43 post TAC surgery. (**A**) IF images of cardiac cryosections isolated from control and *Dsp^R451G/+^* mice 12 weeks post-TAC surgery. Protein targets examined included plakoglobin (**B**) and Cx43. N-cadherin co-stain is used as a control marker. White arrowheads point to increased expression of Cx43 at the ID. Magenta arrowheads point to mislocalization of Cx43 away from the ID. Scale bars represent 10 μm. *n* = 4 per genotype. (**C**) Quantification of PKG/N-Cad (**D**) and Cx43/N-Cad signal intensity ratios. (**E**) Quantification of Cx43/N-Cad area overlap. Stats performed using Student’s *t*-test (*n* = 4 animals per genotype, *n* = 10 IDs analyzed per animal; *** *p* < 0.001).

To determine if external stress may have detrimental effects on the integrity of ID proteins, we performed immunostaining on heart sections isolated post-TAC surgery. Post-TAC heart sections were stained for PKG and Cx43 (Figure 8). Localization of PKG was consistent between *Dsp^R451G^*^/+^ mice and control littermates when compared to the control ID marker N-cadherin (Figure 8A). In control mice, Cx43 displayed minimal changes following TAC surgery. However, *Dsp^R451G^*^/+^ mice displayed disruption in Cx43 localization when compared to N-cadherin, as seen by an apparent punctate localization pattern and less uniform expression at the ID (Figure 8B). As seen at baseline, neither PKG nor Cx43 expression levels at the ID were altered in *Dsp^R451G/+^* mice compared to control mice (Figure 8C,D). Despite no changes in signal strength, the area of the signal overlap between Cx43 and N-cadherin was significantly reduced at the ID in *Dsp^R451G/+^* mice, further supporting more disruption of Cx43 localization in the ID that is exacerbated by stress (Figure 8E). Of note, no changes in Cx43 or pCx43 (S368) expression were identified after TAC surgery via immunoblotting (Supplementary Figure S9). Overall, external stress following TAC surgery negatively impacts the localization of Cx43 at the ID in *Dsp^R451G^*^/+^ mice.

## 4. Discussion

The gap in knowledge on the progression of ACM phenotype and the molecular pathways of ACM pathogenicity makes therapeutic design and preventative treatment challenging. Therefore, developing models that better recapitulate the human phenotype is vital for the progression in ACM research. Our previous study utilized human cardiac biopsy tissues and iPSC-derived cardiomyocytes from an ACM family harboring a pathogenic *DSP* variant (DSP-p.R451G). Biopsy samples from heterozygous p.R451G patients displayed increased fibro-fatty infiltration, decreased DSP expression at the ID, and mislocalization of Cx43 from the ID [11]. Additionally, iPSC-derived cardiomyocytes identified a reduced expression of Cx43 and increased pCx43-S368, a marker associated with reduced channel opening, altered stability, and has been associated with potential downstream protein degradation via the ubiquitin proteolytic system [11,36,37,38,39]. Decreased expression of DSP that was not connected to a loss of *DSP* mRNA was also identified. This post-translational reduction in DSP was ultimately attributed to an increase in calpain sensitivity, which was also linked to additional variants in this N-terminal mutational ‘hotspot’ using both in silico and in vitro techniques [11]. 

Desmoplakin is essential for proper embryonic development and plays a major role in the integrity of both cardiomyocytes and epithelial tissue [40]. Upon breeding heterozygous *Dsp^R451G/+^* mice, no viable *Dsp^R451G/R451G^* mice were detected, suggesting that homozygous expression of the R451G variant is embryonic lethal. Indeed, *Dsp^R451G/R451G^* embryos were only identified in development prior to E10 (Figure 1C,D). While initially surprising, these results were not unexpected, as whole-body knock-out of *Dsp* is embryonic lethal near E6.5 [40]. While we were unable to study *Dsp^R451G/R451G^* mice, the heterozygous whole body *Dsp^R451G/+^* model more accurately portrays the human population, as to date, all familial members in the p.R451G family are heterozygous for the variant [11].

Consistent with the incomplete penetrance found in humans, we observed no basal structural or electrical phenotypic changes in the *Dsp^R451G/+^* mice (Figure 3, Supplementary Figure S3, Supplementary Tables S3 and S4). It is not uncommon for ACM models to lack a severe phenotype at baseline, where external stressors such as drug stimulation or exercise are required for a phenotypic development. Two separate studies used a plakoglobin heterozygous KO model, as well as a transgenic PKP2 p.R735X expressing mouse model. Interestingly, each showed no significant phenotype at six months of age, but following endurance exercise training, these models displayed RV abnormalities, spontaneous arrhythmias, and/or mis-localization of Cx43 [41,42]. 

Both acute and chronic cardiac stressors were used in this study to fully understand the drivers that contribute toward ACM development in *Dsp^R451G/+^* mice. We hypothesized that chronic cardiac stress might contribute to the progression of the ACM phenotype in the *Dsp^R451G/+^* mice. Despite traditionally being considered a RV dominant disease, familial ACM cases with a DSP variant commonly show increased left or biventricular involvement [27], including the p.R451G family [11]. Additionally, LV dysfunction and/or dilation have been previously noted in heterozygous DSP knock-out mice at baseline [13,14]. Therefore, we utilized a pressure overload model to examine the structural and functional changes in the LV of the heart post-TAC. Our model displayed a reduced LV functional output (via fractional shortening %) and a dilated phenotype by 4 weeks post-TAC (Figure 4). Interestingly, ECG recordings displayed frequent fragmented QRS structures as well as inverted T-waves in *Dsp^R451G/+^* mice that were not apparent in control littermates (Supplementary Figure S6). fQRS is a feature reportedly indicative of fibrosis, ischemia, or scaring of the ventricles [43]. Importantly, fQRS has been linked to prolonged QT, and both fQRS and inverted T waves are considered diagnostic parameters for both myocardial scarring and ACM [44,45]. An early ‘concealed’ phase is a significant problem in ACM diagnosis, as individuals that lack any apparent structural cardiac abnormalities are still vulnerable to life-threatening arrhythmias and SCD [3,4,46], a trait that was identified in the p.R451G patients [11]. The *Dsp^R451G/+^* mice lacked a baseline electrical phenotype, however, an increase in prolonged arrhythmic events was noted following catecholaminergic challenge.

Overall, a multitude of pathways contribute to the progression of ACM symptoms [9,47,48]. We aimed to study molecular changes in common ACM-associated proteins with disease development, including PKP2, β-Catenin and Integrin-β1D [25,33,49,50]. We identified no changes in these targets (Supplementary Figure S8). However, similar to what was seen in heart sections in the p.R451G family [11], mild alterations to localization patterns of Cx43 in *Dsp^R451G/+^* mice were identified (Figure 7B,E). While the mislocalization was less significant in our mice, this could be attributed to the lack of a baseline phenotype in our model, as tissues from the human population were collected from an individual diagnosed with ACM following SCD. This is not surprising as Cx43 mislocalization is seen in other ACM models such as an inducible *Pkp2* KO line [17]. Supporting this previous finding, localization patterns of Cx43 were further disrupted in *Dsp^R451G/+^* mice following TAC surgery (Figure 8B,E). Cx43 plays a vital role in gap junction (GJ) formation and function at the ID and has been shown to be recruited to the ID via DSP-EB1 interactions [51]. Desmosomes also stabilize GJ at the disc [52], so the loss of DSP is expected to impact Cx43 localization. While we did not observe fibro-fatty infiltration, this is a feature that is not commonly identified in ACM mouse models. 

We have successfully generated one of the first ACM mouse models that more accurately recapitulates human genetics by introducing the murine equivalent of the human DSP p.R451G variant endogenously. While punctate expression patterns of Cx43 may be linked to arrhythmia formation, *Dsp^R451G/+^* mice displayed less significant mislocalization when compared to human studies. Future studies will focus on how combined external stressors exacerbate this mislocalization, as well as identifying other molecular targets that may only be altered following prolonged stress. In addition, to further elucidate the changes in Cx43 localization, transmission electron microscopy will be performed to determine any changes occurring to gap junctions, as well as other ID structures, that occur in *Dsp^R451G/+^* hearts after the onset of stress. DSP plays a key role in protein recruitment to the ID, as well as providing structural support to the ID/desmosomes [53]. As desmosomes play a vital role in ID stability and membrane integrity, it is also important to consider the effects of this variant on ID stability. While beyond the scope of this initial study, future work will focus on potential morphological the changes in the different ID structures (desmosomes, gap junctions, adherens junctions) with the relationship to ID integrity. 

## Data Availability

Not applicable.

## Supplementary Materials

The following supporting information can be downloaded at: https://www.mdpi.com/article/10.3390/cells11193049/s1, Table S1: Primers; Table S2: Antibodies; Figure S1: Genotyping analysis of the DSP R451G+ mouse model; Figure S2: *Dsp^R451G/+^* mice display a reduction in DSP protein expression at different time points, with no changes in *Dsp* mRNA levels; Table S3: Full list of echocardiographic findings from *Dsp^R451G/+^* mice and control littermates; Figure S3: *Dsp^R451G/+^* display no significant electrical abnormalities baseline; Figure S4: Structural changes in *Dsp^R451G/+^* mice post TAC surgery; Table S4: ECG findings from conscious mice without anesthetic or catecholaminergic stimulation; Figure S5: Surface ECG recordings from *Dsp^R451G/+^* mice and control littermates post-TAC surgery; Figure S6: Representative ECG traces following TAC surgery; Figure S7: Electrocardiographic findings following catecholaminergic stimulation in telemeter mice; Figure S8: No changes in the expression of additional key ID proteins; Figure S9: No changes in expression of Cx43 of pCx43 following TAC surgery.
