# Supplementary material for: Humanized Dsp ACM Mouse Model Displays Stress-Induced Cardiac Electrical and Structural Phenotypes
Source: Cells. 2022 Sep 29;11(19):3049. doi: 10.3390/cells11193049 (PMC9562631; doi:10.3390/cells11193049)
Supplement: Supplementary file 1 [file cells-11-03049-s001.zip › cells-1903435-supplementary.pdf]

Supplementary Material

Supplementary Tables

Table S1: Primers: All primers utilized throughout the manuscript are provided below.

Primer Name	Primer Sequence 5’-3’	Use
***Dsp* R451G Genotyping F**	5’-GCACTCGTGTTAAAGGCAGAC	Mouse Genotyping
***Dsp* R451G Genotyping R**	5’-CCTGGTCTTGTTTGTAGTCGC	Mouse Genotyping
***Dsp* qPCR F**	5’-CACTCTTCAGACGCAGGTGGA	qPCR
***Dsp* qPCR R**	5’-GCCCTTCAGGTAGGCTTCAG	qPCR
***Hprt* qPCR F**	5’-TGCTGACCTGCTGGATTACA	qPCR
***Hprt* qPCR R**	5’-TTATGTCCCCCGTTGACTGA	qPCR

Table S2: Antibodies: Primary and secondary antibodies utilized throughout the manuscript are provided below.

Antibody Target	Manufacturer	Reference	Host	Class	Application	Concentration
**Desmoplakin**	Abcam	ab16434	Mouse	Monoclonal	IB/IF	1:500/1:100
**Desmoplakin**	Abcam	ab71690	Rabbit	Polyclonal	IF	1:100
**Plakoglobin**	Abcam	ab15153	Rabbit	Polyclonal	IB/IF	1:2500/1:100
**Plakophilin-2**	Fitzgerald	10R-P130B	Mouse	Monoclonal	IB/IF	1:1000/1:100
**N-Cadherin**	Invitrogen	33-3900	Mouse	Monoclonal	IB/IF	1:1000/1:200
**N-Cadherin**	Invitrogen	PA5-19486	Rabbit	Polyclonal	IF	1:200
**GAPDH**	Fitzgerald	10R-G109A	Mouse	Monoclonal	IB	1:10000
**Connexin-43**	Abcam	ab11370	Rabbit	Polyclonal	IB/IF	1:2000/1:200
**pConnexin-43 (Ser 368)**	Sigma-Aldrich	AB3841	Rabbit	Polyclonal	IB	1:1000
**Integrin β1D**	Abcam	ab8991	Mouse	Monoclonal	IB	1:1000
**β-Catenin**	Invitrogen	MA1-300	Mouse	Monoclonal	IB	1:1000
**Donkey Anti-mouse HRP (2º)**	Jackson Immunology	715-035-150	Donkey	Polyclonal	IB	1:5000
**Donkey Anti-Rabbit HRP (2º)**	Jackson Immunology	711-035-152	Donkey	Polyclonal	IB	1:10000
**Donkey Anti-Mouse IgG Fluro 488**	ThermoFisher Scientific	A21202	Donkey IgG	Polyclonal	IF	1:400
**Donkey Anti-Mouse IgG Fluro 568**	ThermoFisher Scientific	A10037	Donkey IgG	Polyclonal	IF	1:400
**Donkey Anti-Rabbit IgG Fluro 488**	ThermoFisher Scientific	A21206	Donkey IgG	Polyclonal	IF	1:400
**Donkey Anti-Rabbit IgG Fluro 568**	ThermoFisher Scientific	A10042	Donkey IgG	Polyclonal	IF	1:400

Table S3: Full list of echocardiographic findings from *Dsp*$^{R451G/+}$ mice and control littermates.

Sex	Genotype	IVSd (cm)	LVIDd (cm)	EDV (mL)	LVPWd (cm)	IVSs (cm)	LVIDs (cm)	ESV (mL)	EF%	FS%	LVPWs (cm)
Male	Control	0.04	0.41	0.17	0.05	0.05	0.3	0.07	56.75	25.31	0.07
Male	Control	0.05	0.39	0.15	0.04	0.07	0.28	0.06	60.69	27.71	0.07
Male	Control	0.05	0.35	0.11	0.06	0.06	0.23	0.03	70.75	34.62	0.07
Male	Control	0.04	0.39	0.15	0.06	0.05	0.29	0.06	58.10	26.09	0.06
Male	Control	0.05	0.37	0.13	0.06	0.06	0.28	0.06	57.47	25.68	0.07
Male	Control	0.04	0.42	0.18	0.05	0.05	0.29	0.06	65.82	31.17	0.07
Male	Control	0.04	0.42	0.19	0.05	0.06	0.31	0.08	57.84	26.00	0.06
Female	Control	0.04	0.34	0.1	0.06	0.05	0.26	0.05	53.89	23.50	0.09
Female	Control	0.03	0.34	0.1	0.05	0.05	0.25	0.04	59.76	27.00	0.06
Female	Control	0.04	0.36	0.12	0.05	0.05	0.25	0.04	67.01	31.87	0.06
Female	Control	0.04	0.35	0.11	0.05	0.06	0.23	0.03	70.53	34.45	0.07
Female	Control	0.05	0.35	0.11	0.06	0.06	0.27	0.05	53.51	23.30	0.07
Female	Control	0.04	0.36	0.12	0.05	0.05	0.27	0.05	57.56	25.70	0.06
Female	Control	0.05	0.38	0.14	0.06	0.08	0.25	0.04	68.56	33.04	0.09
	Mean ± SEM	0.0429 ± 0.00163	0.374 ± 0.00760	0.134 ± 0.00796	0.0536 ± 0.00169	0.0571 ± 0.00244	0.269 ± 0.00662	0.0514 ± 0.00390	61.30 ± 1.61	28.25 ± 1.06	0.0693 ± 0.00267
Male	R451G+	0.05	0.42	0.19	0.06	0.07	0.3	0.07	63.49	29.60	0.07
Male	R451G+	0.04	0.39	0.15	0.05	0.06	0.25	0.04	72.31	35.93	0.07
Male	R451G+	0.05	0.42	0.18	0.06	0.06	0.33	0.09	50.53	21.77	0.09
Male	R451G+	0.04	0.39	0.15	0.06	0.05	0.3	0.07	53.49	23.38	0.07
Male	R451G+	0.04	0.35	0.11	0.06	0.05	0.25	0.04	63.04	29.13	0.09
Male	R451G+	0.05	0.36	0.12	0.05	0.06	0.25	0.04	63.89	29.72	0.08
Male	R451G+	0.04	0.35	0.11	0.06	0.06	0.25	0.04	61.33	28.02	0.07
Male	R451G+	0.04	0.39	0.15	0.06	0.05	0.28	0.06	60.98	27.90	0.08
Male	R451G+	0.03	0.37	0.13	0.06	0.06	0.28	0.06	57.11	25.45	0.08
Male	R451G+	0.04	0.4	0.16	0.06	0.05	0.29	0.06	59.05	26.69	0.08
Female	R451G+	0.03	0.37	0.13	0.07	0.06	0.28	0.06	55.78	24.66	0.08
Female	R451G+	0.04	0.39	0.14	0.05	0.05	0.3	0.07	53.28	23.25	0.05
Female	R451G+	0.03	0.37	0.13	0.04	0.05	0.26	0.04	66.04	31.22	0.06
Female	R451G+	0.04	0.37	0.13	0.06	0.05	0.26	0.04	65.80	31.05	0.08
Female	R451G+	0.04	0.38	0.13	0.05	0.05	0.29	0.06	54.22	23.77	0.06
Female	R451G+	0.04	0.36	0.12	0.05	0.05	0.27	0.05	57.56	25.70	0.06
Female	R451G+	0.03	0.39	0.15	0.05	0.07	0.27	0.05	65.70	31.03	0.07
	Mean ± SEM	0.0394 ± 0.00160	0.381 ± 0.00511	0.140 ± 0.00542	0.0559 ± 0.00173	0.0559 ± 0.00173	0.277 ± 0.00554	0.0553 ± 0.00355	60.21 ± 1.40	27.55 ± 0.900	0.0729 ± 0.00268
	p-value	0.145	0.437	0.547	0.353	0.669	0.330	0.470	0.612	0.616	0.346

IVSd/s: Interventricular septum at diastole/systole. LVIDd/s: Left ventricular internal diameter at diastole/systole. EDV/ESV: End diastolic volume/ End systolic volume. LVPWd/s: Left ventricular posterior wall at diastole/systole. EF: Ejection Fraction. FS: Fractional shortening.

Table S4: ECG findings from conscious mice without anesthetic or catecholaminergic stimulation.

Sex	Genotype	RR Interval (s)	Heart Rate (BPM)	PR Interval (s)	P Duration (s)	QRS Interval (s)	QT Interval (s)	QTc (s)	Tpeak Tend Interval (s)
Male	Control	0.0841	715.1	0.0311	0.00830	0.00996	0.0197	0.0215	0.00790
Male	Control	0.105	573.4	0.0316	0.0103	0.0117	0.0202	0.0197	0.00595
Male	Control	0.101	601.8	0.0320	0.00953	0.0108	0.0210	0.0209	0.00815
Male	Control	0.0871	691.7	0.0315	0.00940	0.0110	0.0220	0.0236	0.00990
Female	Control	0.0871	690.0	0.0320	0.00843	0.0112	0.0213	0.0229	0.00675
Female	Control	0.0881	683.8	0.0340	0.00880	0.00987	0.0195	0.0208	0.00664
Female	Control	0.0834	720.5	0.0336	0.00940	0.00942	0.0194	0.0213	0.00872
Female	Control	0.0813	738.3	0.0329	0.00940	0.00998	0.0208	0.0231	0.00682
	Mean ± SEM	0.0896 ± 0.00305	676.8 ± 20.7	0.0323 ± 0.000376	0.00919 ± 0.000229	0.0105 ± 0.000285	0.0205 ± 0.000334	0.0217 ± 0.000473	0.00760 ± 0.000461
Male	R451G+	0.0852	705.7	0.0326	0.0103	0.0101	0.0215	0.0234	0.00972
Male	R451G+	0.0952	635.4	0.0325	0.00848	0.0117	0.0234	0.0240	0.0105
Male	R451G+	0.103	587.4	0.0319	0.00825	0.0117	0.0223	0.0220	0.00834
Male	R451G+	0.0923	655.3	0.0334	0.00868	0.0115	0.0214	0.0223	0.00819
Female	R451G+	0.0836	719.6	0.0327	0.00988	0.0097	0.0208	0.0228	0.00630
Female	R451G+	0.0874	688.5	0.0309	0.00889	0.0098	0.0235	0.0252	0.01173
Female	R451G+	0.0876	688.3	0.0330	0.00876	0.0089	0.0199	0.0213	0.00988
	Mean± SEM	0.0905 ± 0.00251	668.6 ± 17.3	0.032 4± 0.000302	0.00903 ± 0.000285	0.0105 ± 0.000428	0.0218 ± 0.000499	0.0230 ± 0.000493	0.00924 ± 0.000674
	p-value	0.829	0.769	0.859	0.672	0.947	*0.0429	0.0914	0.0609

Supplementary Figures

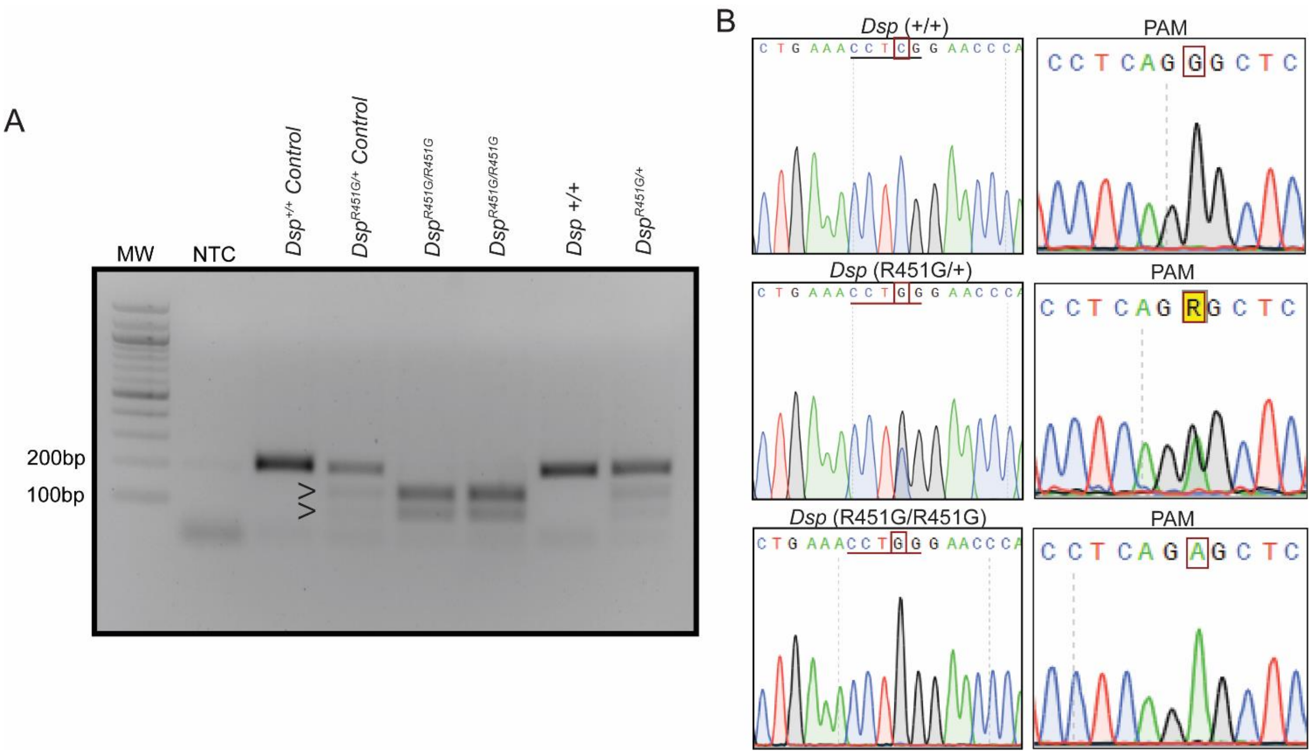

Figure S1: Genotyping analysis of the *Dsp*$^{R451G/+}$ mouse model. **(A)** Representative genotyping of the embryo isolations from *Dsp*$^{R451G/+}$ breeding pairs, with no template control (NTC). Control and R451G+ positive controls previously confirmed with sequencing. Restriction enzyme digest using BstNI cleaves R451G+ DNA, but not control DNA. Control allele is represented by a single band at 192bp. An R451G+ allele is two bands at 103bp and 89bp (arrowheads point to bands). **(B)** Sequencing results verified both heterozygous and homozygous R451G+ mice at the mutation site and via the PAM sequence site.

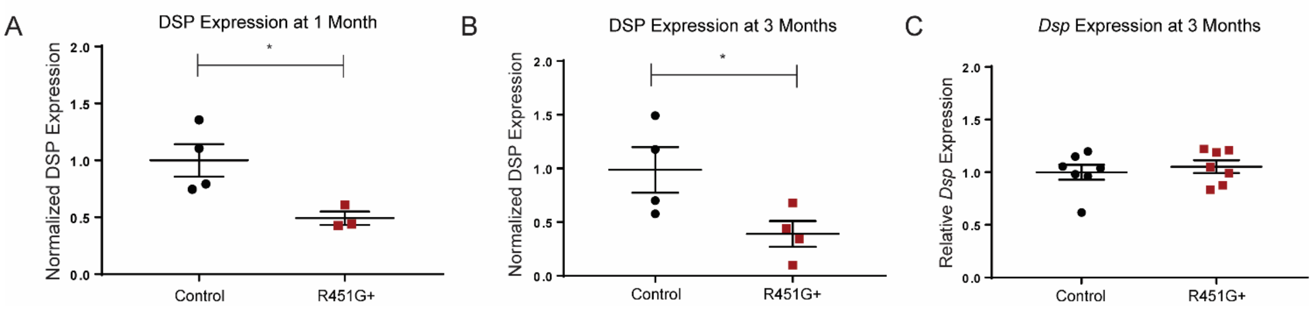

Figure S2: *Dsp*$^{R451G/+}$ mice display a reduction in DSP protein expression at different time points, with no changes in *Dsp* mRNA levels. **(A)** Immunoblot quantification of DSP from heart lysates isolated at 1 month of age (Control n = 4, *Dsp*$^{R451G/+}$ n = 3), **(B)** and 3 months of age (n = 4 per group). **(C)** qPCR examining *Dsp* expression from isolated cDNA from *Dsp*$^{R451G/+}$ and control littermates hearts at 3 months of age, normalized to *Hprt* (n = 7 per group). Stats performed using Student's t-test * $p < 0.05$.

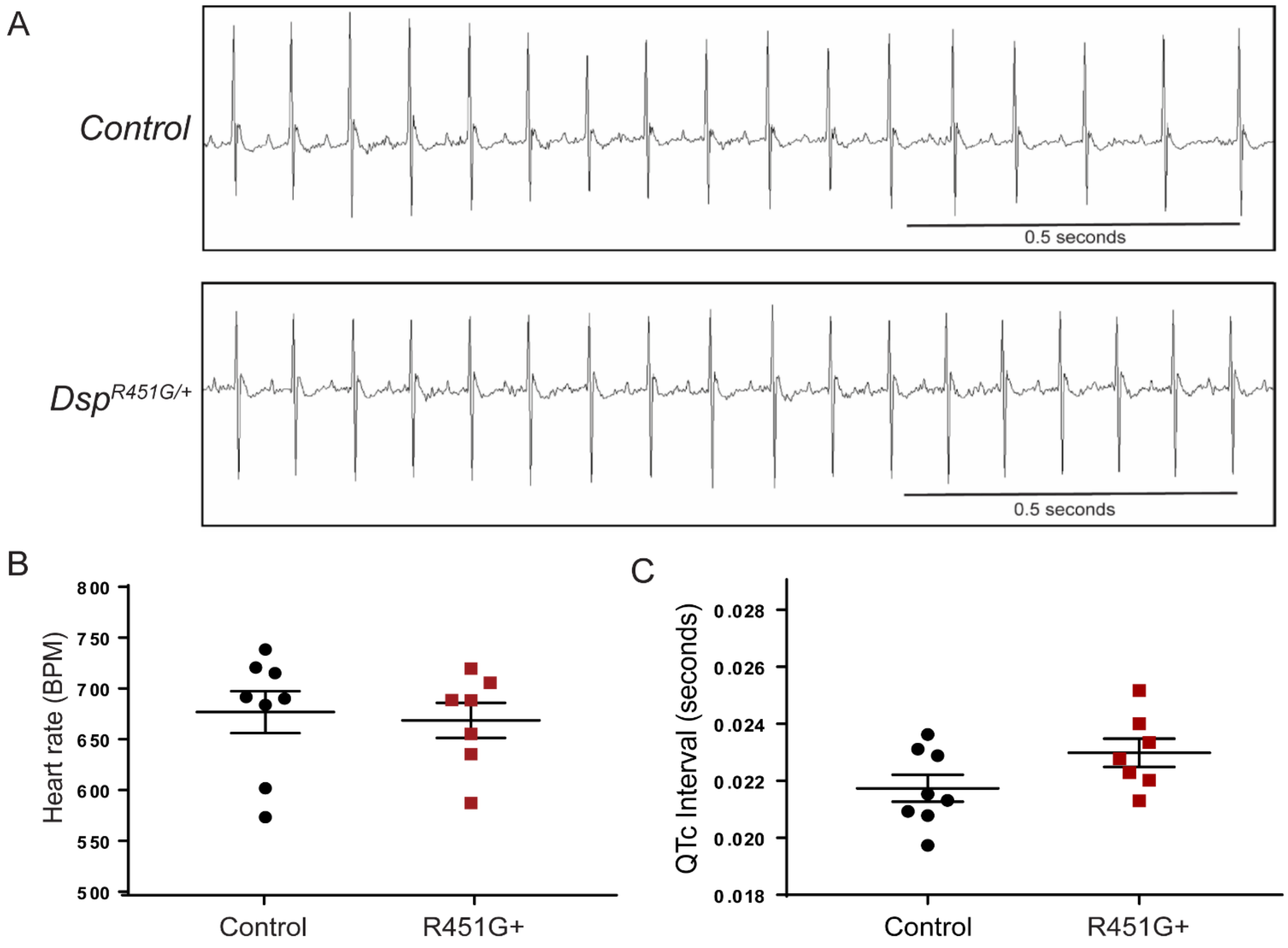

Figure S3: *Dsp*$^{R451G/+}$ display no significant electrical abnormalities baseline. **(A)** Representative ECG trace in conscious mice at ~3 months of age displayed no signs of cardiac arrhythmias or abnormal ECG morphology. Scale bars represent 500ms. **(B)** Heart rate **(C)** and QTc (Mitchell et al. normalization) intervals calculated from telemetry mice at baseline. Stats performed using Student's t-test (control $n = 8$, *Dsp*$^{R451G/+}$ $n = 7$).

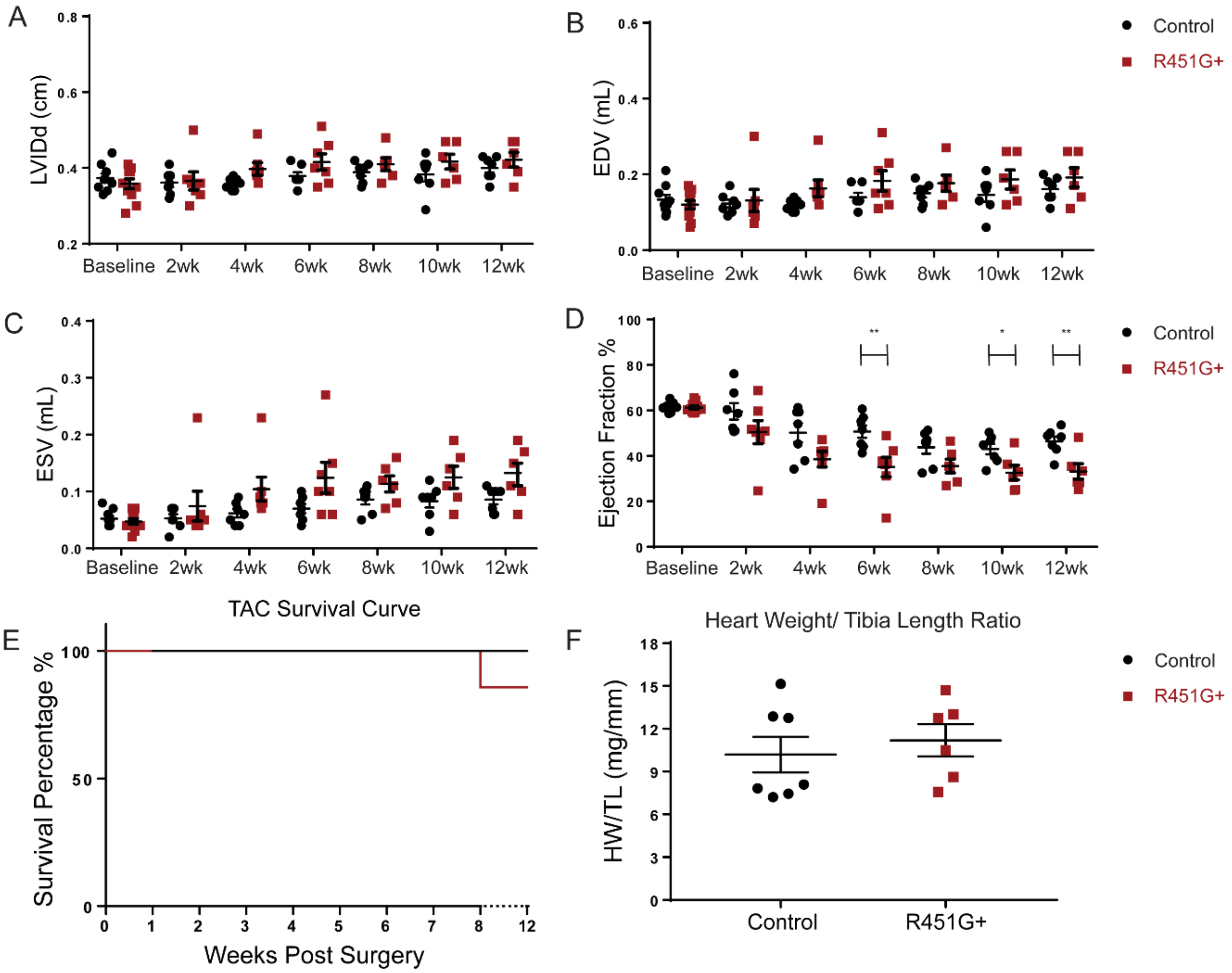

Figure S4: Structural changes in *Dsp*$^{R451G/+}$ mice post TAC surgery. Quantification of additional LV parameters from *Dsp*$^{R451G/+}$ and control littermates following TAC surgeries, including **(A)** left ventricular internal diameter at diastole, **(B)** end-diastolic volume, **(C)** end-systolic volume, **(D)** and ejection fraction. **(E)** Kaplan–Meier curve following TAC surgery. **(F)** Heart-weight-to-tibia length ratio at endpoint experiments/heart isolations. (control $n = 7$, *Dsp*$^{R451G/+}$ $n = 6\text{-}7$ post-surgery). Stats performed using Student's t-test at each timepoint * $p < 0.05$, ** $p < 0.01$.

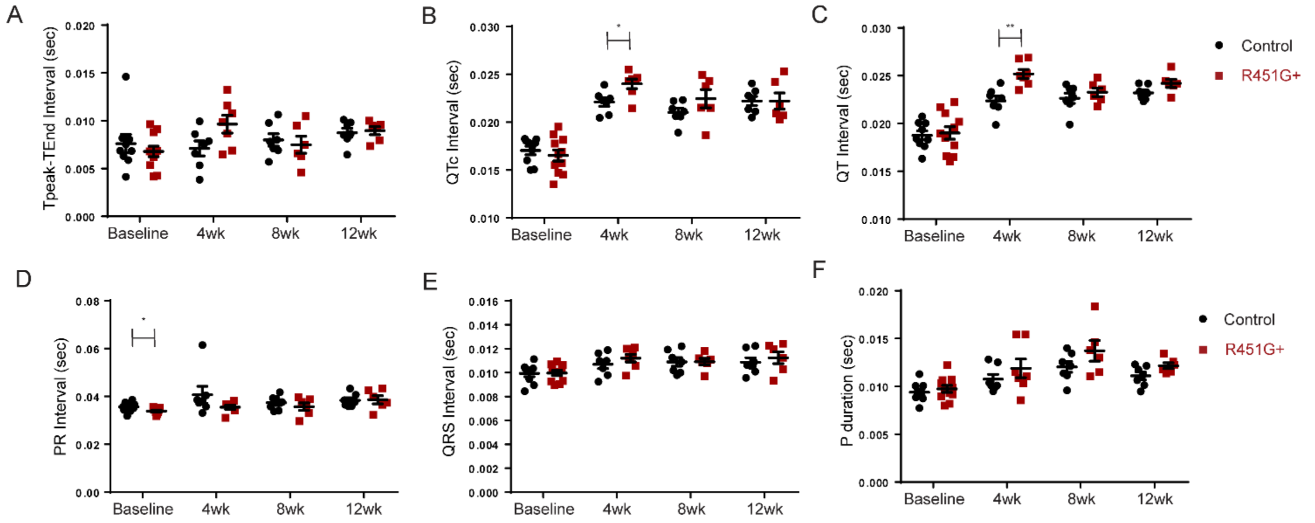

Figure S5: Surface ECG recordings from $Dsp^{R451G/+}$ mice and control littermates post-TAC surgery. Parameters analyzed include **(A)** T-peak to T-end duration, **(B)** QTc duration (Mitchell et al. normalization), **(C)** QT interval, **(D)** PR interval, **(E)** QRS interval, **(F)** and p duration. Stats analyzed using Student's t-test at each time point, * $p < 0.05$ ** $p < 0.01$ (control $n = 7$, $Dsp^{R451G/+}$ $n = 6$-7 post-surgery).

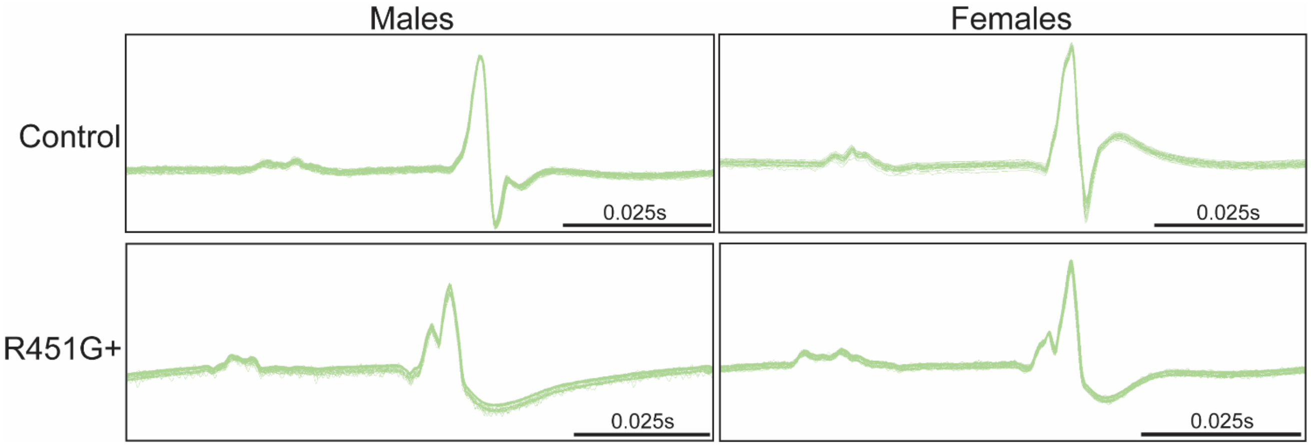

Figure S6: Representative ECG traces following TAC surgery. Representative traces are average ECG morphology of *Dsp*$^{R451G/+}$ mice and control littermates at 4 weeks post-TAC. Scale bars represent 0.025 seconds.

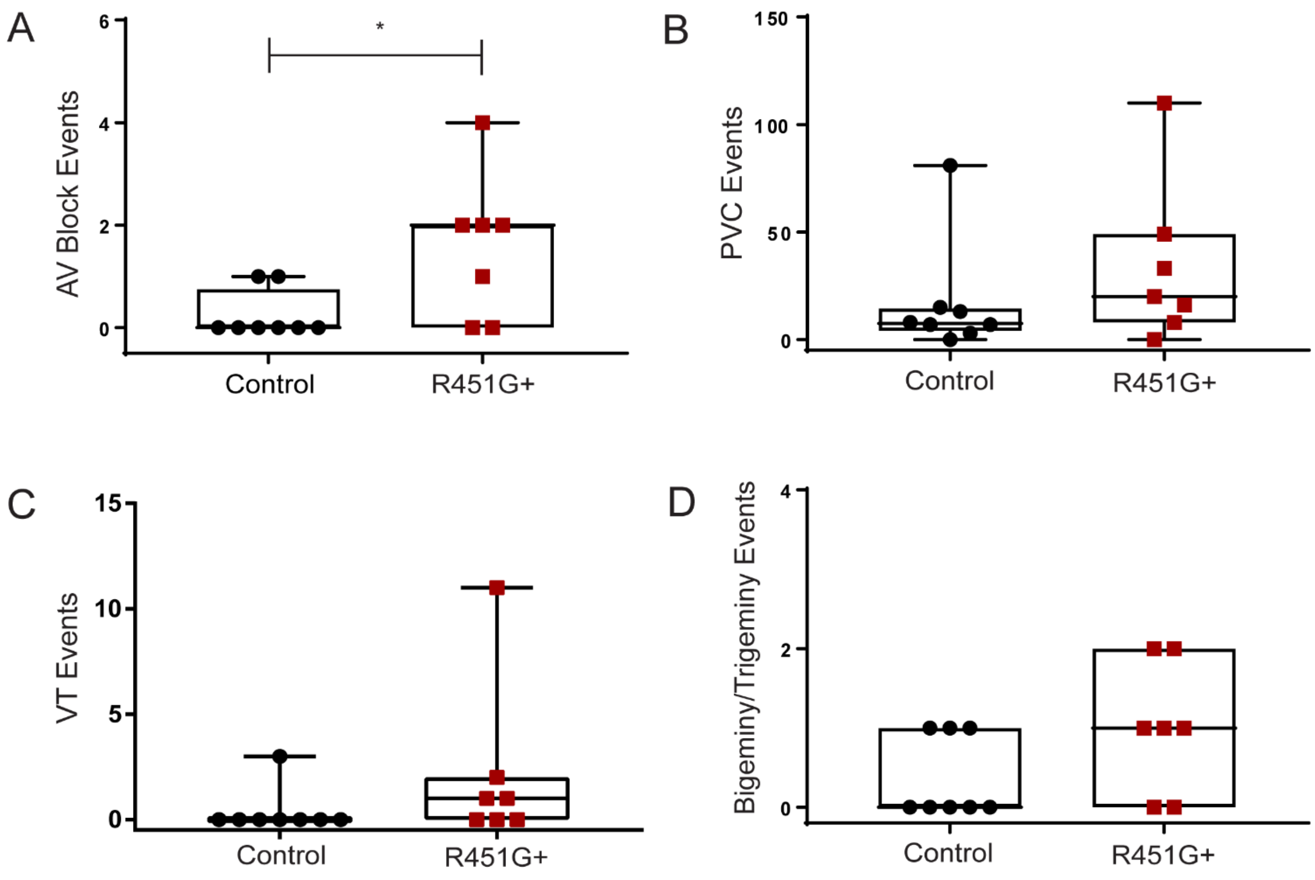

Figure S7: Electrocardiographic findings following catecholaminergic stimulation in telemeter mice. **(A)** Breakdown of different arrhythmia events in mice following epinephrine injections, including atrioventricular block, **(B)** premature ventricular contractions, **(C)** ventricular tachycardia, **(D)** and bi/trigeminy. Stats performed using Mann–Whitney non-parametric test. * $p < 0.05$ (control $n = 8$, $Dsp^{R451G/+}$ $n = 7$).

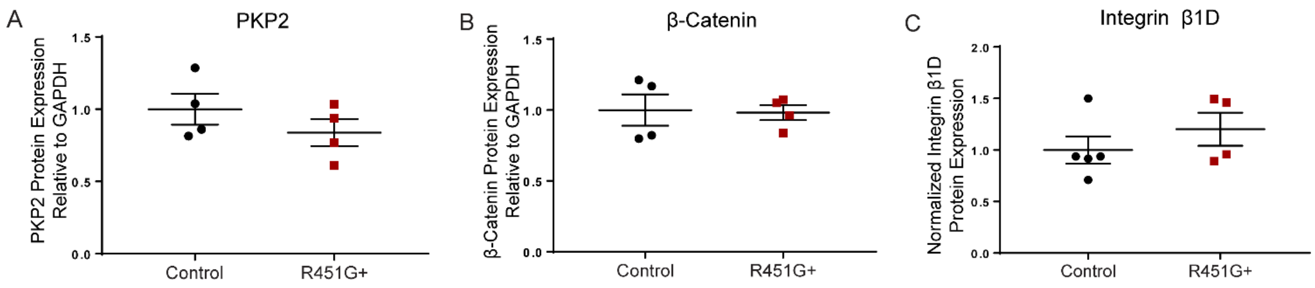

Figure S8: No changes in the expression of additional key ID proteins. Immunoblots of heart lysates from *Dsp*$^{R451G/+}$ and control littermates isolated at 3 months of age, evaluating expression of **(A)** plakophilin-2 **(B)** and β-catenin (*n* = 4 per genotype). Quantification of each target relative to appropriate GAPDH staining. Note β-catenin shares the same GAPDH as the blots on figure 6 as it was probed for on the same blot. **(C)** Immunoblot probing for Integrin β1D (control *n* = 5, *Dsp*$^{R451G/+}$ *n* = 4) quantified, relative to Ponceau staining. Stats performed using Student's t-test.

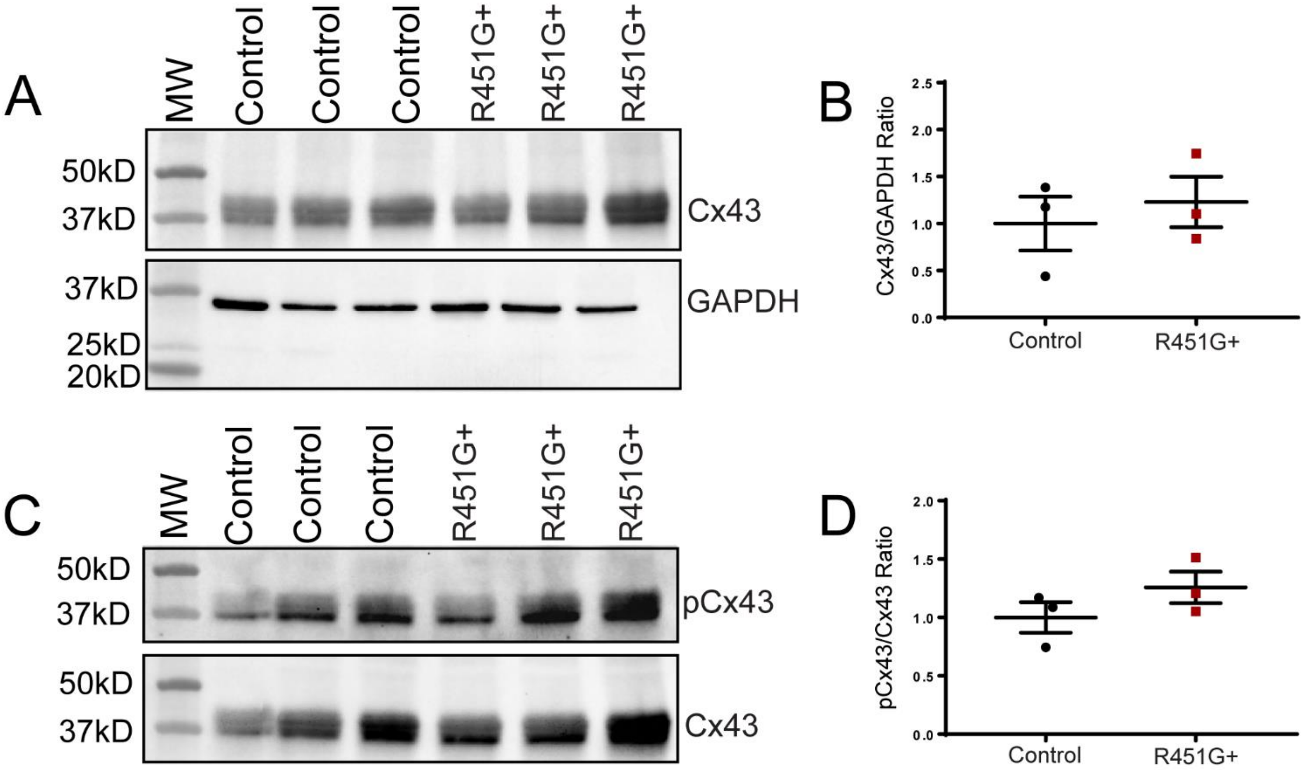

Figure S9: No changes in expression of Cx43 of pCx43 following TAC surgery. **(A)** Immunoblot probing for Cx43 and GAPDH **(B)** with quantification. (C) Immunoblot probing for S368-pCx43 and associated Cx43 staining (D) with quantification. Stats performed using Student's t-test (n = 3 per genotype).
